# A Case Report of Adrenocorticotropic Hormone to Treat Recurrent Focal Segmental Glomerular Sclerosis Post-Transplantation and Biomarker Monitoring

**DOI:** 10.3389/fmed.2015.00013

**Published:** 2015-03-20

**Authors:** Siddiq Anwar, Derek S. Larson, Nima Naimi, Muhammad Ashraf, Nancy Culiberk, Helen Liapis, Changli Wei, Jochen Reiser, Daniel C. Brennan

**Affiliations:** ^1^Department of Medicine, Washington University School of Medicine, St. Louis, MO, USA; ^2^Department of Pathology, Washington University School of Medicine, St. Louis, MO, USA; ^3^Department of Medicine, Rush University Medical Center, Chicago, IL, USA

**Keywords:** acute kidney injury, albumin permeability factor, angiotensin 1 receptor antibody, podocyte, recurrent focal segmental glomerular sclerosis, soluble urokinase plasminogen activator receptor

## Abstract

**Background:** Recurrent focal segmental glomerular sclerosis (rFSGS) in renal transplant recipients (RTR) is difficult to predict and treat. Early rFSGS is likely from circulating factors and preformed antibodies.

**Methods:** We present the case of a 23-year-old white man who presented with rFSGS and acute renal failure, requiring dialysis 9-months after a 1-haplotype matched living-related transplant. We retrospectively analyzed serum samples from various clinical stages for rFSGS biomarkers: serum glomerular albumin permeability (P_alb_), soluble urokinase-type plasminogen activator receptor (suPAR) serum level with suPAR-β3 integrin signaling on human podocytes, and angiotensin II type I receptor-antibody (AT1R-Ab) titer.

**Results:** All biomarkers were abnormal at 1-year pre-transplant prior to initiation of dialysis and at the time of transplant. After initiation of hemodialysis, β3 integrin activity on human podocytes, in response to patient serum, as well as AT1R-Ab were further elevated. At the time of biopsy-proven recurrence, all biomarkers were abnormally high. One week after therapy with aborted plasmapheresis (secondary to intolerance), and high dose steroids, the P_alb_ and suPAR-β3 integrin activity remained significantly positive. After 12-weeks of treatment with high-dose steroids, rituximab, and galactose, the patient remained hemodialysis-dependent. Three-months after his initial presentation, we commenced adrenocorticotropic hormone (ACTH, Acthar^®^ Gel), 80 units subcutaneously twice weekly. Four-weeks later, he was able to discontinue dialysis. After 8-months of maintenance ACTH therapy, his serum creatinine stabilized at 1.79 mg/dL with <1 g of proteinuria.

**Conclusion:** ACTH therapy was associated with improvement in renal function within 4 weeks. The use of rFSGS biomarkers may aid in predicting development of rFSGS.

## Introduction

Renal allograft loss due to recurrence of idiopathic focal segmental glomerular sclerosis (rFSGS) is common, with an incidence of 30–50% (1–3). Recurrent FSGS has been reported within 1 week after transplant (4); and it has been hypothesized that a circulating factor is responsible (5, 6). Injecting laboratory animals with serum from patients with FSGS caused proteinuria and glomerular lesions reminiscent of FSGS (7). Serum from FSGS patients induced increased glomerular albumin permeability (P_alb_) in isolated glomeruli *ex vivo* (5). Savin et al. suggested that the permeability factor is a 50 kD plasma protein (5), but the nature of this P_alb_ factor is still undefined. The approximate 50 kD sized serum soluble urokinase receptor (suPAR) is one of the leading candidates. Elevated serum levels of circulating suPAR (more than 3000 pg/dL) have been proposed to confer heightened risk for rFSGS by inducing podocyte injury through activation of β3-integrin (8). A recent report suggested antibodies to the angiotensin II type 1 receptor (AT1R-Ab) may contribute to development of rFSGS in renal transplant recipients (RTR) by causing podocyte injury and severe podocyte foot process effacement (9).

Our patient presented with rFSGS. Therefore, we analyzed three biomarkers in parallel and measured serum suPAR and the suPAR beta3-integrin axis after incubation of human podocytes with the patient’s sera, P_alb_, and AT1R-Ab serially in samples obtained 1-year before transplant, at the time of transplant, when he was admitted with rFSGS and acute kidney injury 9-months after transplantation and 1- and 12-weeks after treatment.

Our patient did not respond or could not tolerate usual methods of treatment for rFSGS. Adrenocorticotropic hormone Acthar^®^ (Acthar Gel, Questcor, Anaheim Hills, CA, USA) has been shown to induce complete or partial remission in a percentage of patients with treatment-resistant FSGS in native kidneys, with 2 of 5 responders in one study and 8 of 24 responders in another (10, 11). We report the use of Acthar in this patient with rFSGS.

## Subjects and Methods

This study was approved by the Human Research Protection Office (HRPO) of Washington University School of Medicine. A 23-year-old white male with a history of end stage renal disease (ESRD) secondary to FSGS presented 9-months after undergoing a one-haplotype matched renal transplant on 24 July 2012 from his father with a low-grade fever consistent with a non-descript viral illness, malaise, progressive edema, acute kidney injury, and nephrotic range proteinuria. The complement dependent cytotoxicity (CDC) and flow cross-match were negative pre-transplant.

At the time of his transplant surgery, he had received thymoglobulin 5 mg/kg over 3 days and was maintained on tacrolimus, enteric coated mycophenolic acid (MPA), and prednisone. There were no preformed donor specific antibodies. He developed low-level cytomegalovirus (CMV)-viremia (Table 1) and leukopenia 4-months after transplantation, and his MPA was discontinued and the valganciclovir (VGCV) dose increased from 450 to 900 mg daily for treatment. Tacrolimus levels ranged 4–7 ng/mL. His CMV-viremia resolved within 4 weeks (Table 1) and he was treated with consolidation therapy with VGCV, 900 mg daily, which he was still taking at presentation.

**Table 1:** **Time course of clinical events, laboratory results and biomarkers**.

	1 year pre-transplant	At transplant	1 month post-transplant	3 months post-transplant	4 months post-transplant	9 months post-transplant (admitted with AKI)	1 week after admission (after pulse steroids and one PE)	12 months post-transplant (2 weeks before starting ACTH)	15 months post-transplant (2.5 months on ACTH)	18 months post-transplant (5 months on ACTH)	28 months post-Transplant (8 months after ACTH therapy)
Creatinine (mg/dL)	4.09	HD	1.3	1.2	1.2	HD	HD	HD	2.9	2.3	1.83
FK trough (ng/mL)	–	–	6.4	6	5.8	–	–	5.6	3.4	1.6	2.4
CMV PCR (copies/mL)	–	–	–	4533	<200	<200	–	–	ND	ND	ND
BK virus PCR	–	–	–	ND	–	ND	–	–	ND	ND	ND
Albumin (g/dL)	4.9	4.5	4.7	4.9	4.7	3.1	2.5	2.6	3.6	4.4	4.8
P/C ratio	–	–	0.09	–	–	17.7	–	–	7.2	2.2	0.34
DSA	–	ND	–	ND	–	ND	–	ND	–	–	–
P_alb_	0.77	0.51	–	–	–	0.61	0.58	0.43	–	–	–
SuPAR (pg/mL)	3573	2898	–	–	–	4691	4428	3447.8	–	–	–
AT1R antibody (U/mL)	20.4	23.2	–	–	–	16.8	13.5	9.3	–	–	–

*AKI, acute kidney injury; HD, hemodialysis; CMV, cytomegalovirus; IgG, immunoglobulin G; PCR, polymerase chain reaction; PE, plasma exchange; ND, not detected; FK, tacrolimus; P/C, protein to creatinine ratio; DSA, donor specific antibodies; NA, not applicable; suPAR, soluble urokinase receptor*.

*Suspicious: >3000 pg/mL, values >3500 pg/mL are suggestive of suPAR-mediated FSGS (Dr. Reiser’s Lab, Rush University, USA). P_alb_: glomerular albumin permeability factor. Normal: 0. Nonspecific: 0.2–0.5. Increased risk: >0.5 (Renal Research, KC VA Medical Center, Kansas City, MO, USA). Angiotension-1 receptor antibody: AT1Rab. Negative: <10. Borderline: 10–17. Positive: >17 (Immunogenetics Laboratory, The Johns Hopkins University, Baltimore, MD, USA)*.

## Results

Nine-months after his renal transplant, he presented with elevated serum creatinine from a baseline of 1.2–2.5 mg/dL. His 24-h urine protein excretion revealed 10 g of proteinuria. There were no donor specific antibodies identified by single antigen bead assay (Luminex, One-lambda, Los Angeles, CA, USA). His blood CMV-PCR was negative, and he had a normal renal transplant sonogram.

**Figure 1:**
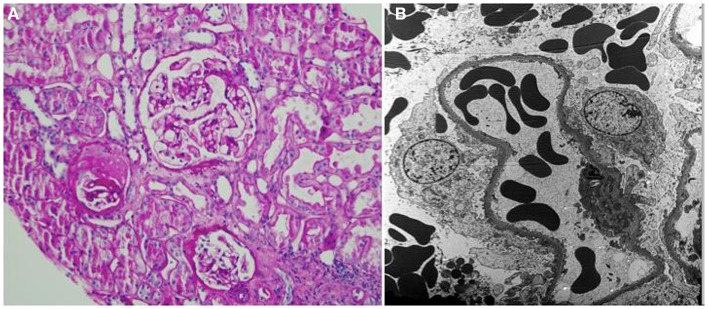
**(A)** Periodic acid-Schiff stain shows one partially sclerotic glomerulus and two intact glomeruli; there is no tubulitis or interstitial fibrosis; minor tubular epithelial cell changes are noted (×200). **(B)** Electron microscopy shows foot process effacement (approximately 30% of the surface area) and reactive cytoplasmic changes in podocytes. Numerous red blood cells are present in the capillary loops and Bowman’s space (×3000).

A renal allograft biopsy demonstrated 19 glomeruli, one of which was partially sclerosed and one was globally sclerosed (Figure 1A). There was one medium size artery with no arteritis. There was moderate arteriolar thickening. There was no tubulitis. Periodic acid-Schiff and trichrome stains confirmed acute tubular injury and highlighted minimal interstitial fibrosis. There was no evidence of thrombotic microangiopathy. Immunofluorescence staining was negative for C4d in peritubular capillaries. There was no peritubular capillaritis. Electron microscopy showed extensive foot process effacement and detached podocytes with reactive cytoplasmic changes and villiform transformation (Figure 1B). Abundant red blood cells (RBCs) were noted in the Bowman’s space. The findings were consistent with rFSGS.

We analyzed stored blood samples from the Washington University Kidney Translational Research Core (NIH/NIDDK P30 DK079333) and tested for glomerular P_alb_ (Savin Lab, Renal Research, Kansas City Veterans Administration Medical Center, Kansas City, MO, USA), suPAR, suPAR-beta3 integrin (β3 integrin) signaling (Reiser Lab, U of ILL at Chicago, Chicago, IL, USA), and angiotensin II type I receptor-antibody (AT1R-Ab) (Immunogenetics Laboratory, Johns Hopkins University, Baltimore, MD, USA) on samples from 1-year pre-transplant, at the time of transplant, and when the patient presented with acute kidney injury (Table 1). To overcome a possible confounder of elevated serum suPAR in the setting of reduced GFR, we also evaluated the activity of β3 integrin signaling on cultured human podocytes incubated with the patient’s sera. With this assay, strong integrin activation is indicative of the presence of pathologic suPAR forms, but not physiologic or inflammatory suPAR that accumulated from decreased clearance (12).

The levels of suPAR, suPAR-β3 integrin activation, and P_alb_ were elevated prior to transplant (3573 pg/mL, 1.63, and 0.77, respectively) and at the time of recurrence (4691 pg/mL, 1.99, and 0.61, respectively). suPAR values >3500 pg/mL and β3 integrin activity >1.3–1.5 are suggestive of suPAR-β3 integrin mediated FSGS. A P_alb_ of 0 is normal and values >0.5 designates an increased risk (non-specific if between 0.2 and 0.5). The level of AT1R-Ab was also increased prior to transplant, 20.4 U/mL, but was borderline normal at 16.8 U/mL at the time of recurrence. AT1R-Ab is presumed to be negative when <10, borderline when 10–17, and positive when >17 (Table 2).

**Table 2:** **Interpretation of putative markers in FSGS**.

Soluble urokinase receptor: suPAR	Normal: undetermined	Intermediate risk: >3000 pg/mL	Suggestive of suPAR-mediated FSGS: >3500 pg/mL
Relative podocyte β3 integrin activity/paxillin on human podocytes compared to control (10% patient sera)	<1	Intermediate risk: >1	Highly suggestive of suPAR- β3 integrin mediated FSGS: >1.5
Glomerular albumin permeability factor: P_alb_	Normal: 0	Non-specific: 0.2–0.5	Increased risk: >0.5
Angiotension-1 receptor antibody: AT1Rab	Negative: <10 U/mL	Borderline: 10–17 U/mL	Positive: >17 U/mL

**Figure 2:**
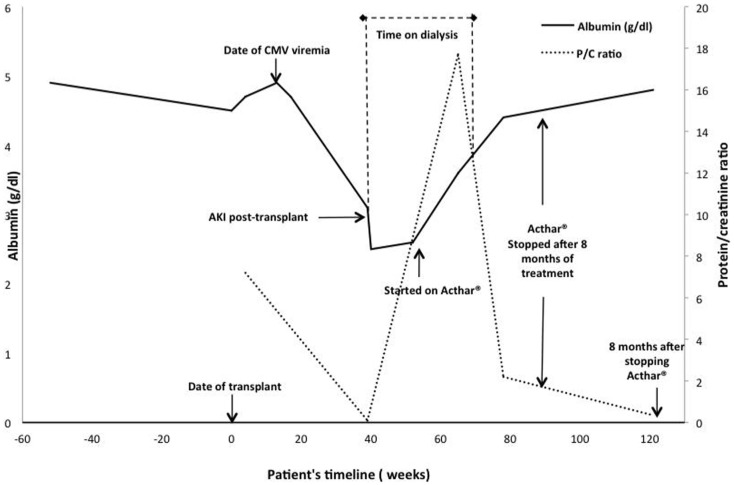
**Time course of events, treatments, and responses in serum albumin and urine protein excretion**. The serum albumin level over time is depicted by the solid black line and the urine protein/creatinine ratio by the dashed gray line. The value at 40 weeks was imputed to be 0 because the patient was anuric.

The patient was started on methylprednisolone 500 mg daily for three days (Figure 2). Plasmapheresis with 1.5 plasma volume exchanges with fresh frozen plasma followed by 10 g of IVIG the day of his renal biopsy was attempted. However, shortly after commencement, the patient developed wheezing, shortness of breath, and an erythematous rash on his trunk, and extremities suggestive of an allergic reaction, and plasmapheresis was discontinued. The next day we attempted plasmapheresis with albumin, and again the patient developed similar symptoms. We considered plasmapheresis with a hypoallergenic dialyzer column, but he refused this treatment. We administered rituximab 200 mg intravenously, once. This low-dose was chosen because of our experience with efficacy with this dose for other recurrent glomerulonephritidies and others have reported that even 100 mg can successfully treat recurrent FSGS (13–16).

Five-days after the initial presentation, the patient became anuric and he required hemodialysis (Figure 2). We initiated lisinopril and arranged for outpatient hemodialysis. We attempted to treat him with galactose (15 g of galactose twice a day for 6 weeks) in view of previously published data on possible benefit (17, 18). Unfortunately, the galactose had to be discontinued after 4-weeks due to severe diarrhea. He subsequently received oral prednisone 100 mg daily as he had responded to a similar dose during his previous four FSGS recurrences as a child prior to his renal transplantation. At the end of 8-weeks, the patient was still hemodialysis-dependent and the prednisone was gradually tapered to 10 mg daily over 2 months. We reassessed the three circulatory factors. His P_alb_ was 0.43 (0 is normal), his suPAR was 3447.9 pg/mL (negative < 3000 pg/mL, β3 integrin activity was not available due to sample unavailability), and AT1R-Ab level was 9.3 U/mL (negative < 10 U/mL).

Due to the significant side effects of prednisone, persistent renal failure, and lack of efficacy, we initiated ACTH treatment (Acthar^®^ Gel,), 80 units twice weekly at 3.5-months after his initial presentation (11). One-month after initiation of ACTH treatment, the urine output increased, creatinine improved, and the patient was able to discontinue dialysis. After 8-months on this therapy, his creatinine decreased and remained stable at 2.0–2.5 mg/dL. His serum albumin improved from 2.6 to 4.6 g/dL and his urine protein-creatinine ratio decreased from 17.7 to 1.2. The ACTH was discontinued. At last follow up, 28 months after transplant and 8 months after discontinuation of ACTH, the serum creatinine was 1.83 mg/dL, and a urine protein creatinine ratio was 0.34, and his serum albumin is 4.8 mg/dL (Figure 2).

## Discussion

Various factors have been implicated as etiologic for rFSGS and include suPAR (8), P_alb_ (5), and AT1R-Ab (9). This case documents rFSGS in a renal transplant recipient in whom the levels of suPAR- β3 integrin signaling, P_alb_, and AT1R-Ab were measured in combination and serially; starting from the pre-transplant period through recurrence and follow up. At the time of transplant, the P_alb_ and AT1R-Ab and podocyte β3 integrin activity were elevated, which means that podocyte β3 integrin was elevated in focal adhesions (represented by paxillin staining), the contact points of podocytes with the extracellular matrix (12). These biomarkers identified the patients as high risk for rFSGS despite the fact that he remained recurrence free for the first 9-months following his transplant. It will require further studies to understand why the patient was disease free for the first 9-months after transplant. The use of rabbit anti-thymocyte globulin (rATG) for induction may have contributed as rATG has been associated with less rFSGS (19). Of note, rabbit-ATG is no longer detectable in blood 6–9 months after administration – the time of the recurrence in the face of persistently high putative biomarkers for rFSGS (20).

The role of suPAR in proteinuric renal disease is currently under active investigation (21). It may be possible that suPAR plays a role in both genetic and non-genetic forms of rFSGS (9); even so rFSGS is generally less prominent in genetic forms of FSGS (8). Some reports show inconsistencies in associations of suPAR and rFSGS in patients with advanced CKD and reduced eGFR in pediatric (22, 23) and adult FSGS patients (24).

Some of the inconsistencies between reports may be related to the quality of the stored samples and the availability of all relevant clinical information including renal biopsy. It is also likely that different suPAR fragments exist and that all of those may not be readily measured with current ELISA, and thus the need to detect the activating capacity of suPAR at the level of the podocyte. In addition, urinary suPAR was recently shown to be highly specific for FSGS and FSGS-urine strongly activated β3 integrin on human podocytes (25, 26). In the future, urinary suPAR measurements in urine may provide valuable information in assaying the suPAR forms that are most relevant to FSGS.

Savin et al. reported a method to test for increased glomerular capillary permeability after incubation with potentially injurious agents or with medium containing 2% vol/vol patient serum. The permeability activity (termed P_alb_ activity or P_alb_) is used to infer the presence of a circulating factor. P_alb_ is calculated from the ratio of capillary expansion of experimental and control glomeruli. Values range from 0, indicating no activity, to 1, indicating complete loss of the permeability barrier. Values >0.5 have been associated with a high incidence of rFSGS following RTR (5). This permeability factor has a high affinity for galactose and P_alb_ activity was lost after galactose infusion in a RTR with rFSGS (17).

Our patient had elevated AT-1R Abs throughout his course. Podocytes express AT-1R (27, 28), and antibodies against the AT1R have been hypothesized to play a role in many kidney diseases. They have been shown to be elevated in vascular rejection (29), pre-eclampsia (30), and systemic sclerosis (31). The elevated levels suggest a possible role of AT1R-Ab in podocyte injury. In one case report of rFSGS, AT1R-Ab level was elevated and the patient was treated successfully with plasmapheresis and angiotensin converting enzyme inhibitors (ACEI) (9). While the clinical improvement of the patient after plasmapheresis suggests removal of antibodies, it does not prove the pathogenicity of AT1R-Ab in this case.

Our report provides valuable insights into the use of ACTH to treat rFSGS. Adrenocorticotropic hormone is a drug which has been approved for infantile spasms, but also carries the indication for nephrotic syndrome (32). Its use in renal transplant patients has not been reported for recurrent nephrotic syndrome. Although not completely defined, ACTH, either alone or via its breakdown product α-melanocyte-stimulating hormone, may induce a potent anti-inflammatory effect by reducing B- and T-cell activity and may also have a direct, podocyte-sparing effect within the glomerulus (33). This would be consistent with the observation that serum suPAR and the integrin activation profile were decreasing. We were able to discontinue the ACTH after 8-months of therapy without evidence of recurrence 8-months after discontinuation.

There are several limitations to our study. The study is retrospective and based on stored serum samples. As such, some level of sample deterioration is possible. We also did not have sufficient serum to assess our complete biomarker panel between transplant and recurrence and we did not have samples for biomarker assessment at the time of ACTH effectiveness. Lastly, we cannot validate the sensitivity, specificity, and variability of these assays.

In conclusion, this case of rFSGS suggests that elevated suPAR-β3 integrin signaling, P_alb_, and AT1rab levels at pre-transplant confer an increased risk of developing rFSGS. This risk assessment is consistent with previously reported studies (12). However, the case also demonstrates the difficulty in understanding the precise cause as well as the course of rFSGS. Our patient developed recurrence 9-months later when serum suPAR increased by more than 50%. This could have been the initiating injury that caused a point of no return for podocyte injury.

Measuring suPAR and P_alb_ levels in young transplant recipients with a history of FSGS in their native kidneys and/or when planning for living transplants might provide additional information to develop immunosuppression strategies and to optimize post-transplant monitoring.

Adrenocorticotropic hormone therapy provides transplant physicians with an additional therapeutic option for proteinuric kidney disease. Given the success in this case, the relationship of adrenocorticotropic hormone therapy and suPAR needs to be better defined on a molecular level as well as in controlled trials, possibly as an *ad hoc* to the trial currently underway for transplant glomerulopathy (34).

## Conflict of Interest Statement

Jochen Reiser is the inventor on pending and issued patents on novel anti-proteinuric therapies and technologies. He stands to gain royalties from their commercialization. All other authors declare that they have no relevant financial interests. The results of this paper have not been published previously in whole or part, except in abstract form. It is not under consideration for publication elsewhere.
